# Supplementary material for: Architecture of an asymmetric short chain/long chain hybrid acyl‑CoA carboxylase from Mycobacterium smegmatis
Source: Commun Biol. 2026 Jun 11;9:1251. doi: 10.1038/s42003-026-10439-x (PMC13612425; doi:10.1038/s42003-026-10439-x)
Supplement: Supplementary file 1 [file 42003_2026_10439_MOESM1_ESM.pdf]

nature portfolio

Peer Review File

Architecture of an asymmetric short chain/long chain hybrid acyl-CoA carboxylase from Mycobacterium smegmatis

Corresponding Author: Professor Matthias Wilmanns

This file contains all reviewer reports in order by version, followed by all author rebuttals in order by version.

Version 0:

Reviewer comments:

Reviewer #1

(Remarks to the Author)
The article "Architecture of an asymmetric short chain/long chain hybrid acyl-CoA carboxylase" is a unique and important addition to the field of carboxyltransferase structure-function. The authors determine structures of a Mycobacterial acyl-CoA carboxylase enzyme complex. This complex is unique compared to ones previously solved due to asymmetry in three senses, i) asymmetry in the typically homohexameric carboxyltransferase (CT) core whereby three short-chain carboxyltransferase dimers have one dimer replaced by a long-chain carboxyltransferase dimer, ii) the trimeric biotin-carboxylase (BC)/biotin carboxy carrier protein (BCCP) is replaced by tetrameric versions, iii) a common 8-stranded beta-barrel with a central alpha-helix that interfaces between the BC and CT is truncated to two beta-sheets from each BC/BCCP but includes a peptide that comprises the central alpha-helix and contributes one strand to the beta-barrel. The arrangement leads to an exaggerated asymmetry. This structure helps resolve a mystery for why there are multiple carboxyltransferase subunits in Mycobacteria. This structure also raises many questions beyond the mycobacterial system. In other Actinobacteria, there appear to be orphan carboxyltransferase domains, could this AccA3/D4/D5/E5 asymmetric system in fact represent a majority of Actinobacterial carboxyltransferase systems? Has the field been missing these asymmetric complexes due to an insistence of using heterologous expression? While this manuscript does not directly address these questions, it will inspire the field to reassess the study of carboxyltransferase complexes using native expression.

I don't see any major issues with the data collection or processing statistics and the authors don't appear to be overinterpreting their data. The most important questions being addressed are the stoichiometry and overall composition of the complex, which is interpretable from the multiple data sets collected. A more thorough characterization of the complex would make the paper more satisfying, such as, what are the relative carboxylation rates for SC vs LC substrates? However, such experiments are not needed to justify the reported structures.

I only have questions that the authors could speculate on for why such a complex might exist.

Does the ratio of AccD4 to AccD5 in the complex suggest that the enzyme is balancing the production of SC and LC malonyl-CoAs towards the SCs?

Do the authors think there could be shuttling of carboxyl-groups between the SC and LC active sites? Could this be a role for the "extra" BC/BCCPs in the complex?

Due to errors in the .pdf provided the methods for Bacterial strain and growth conditions and Mass spectrometry are missing and can't be evaluated.

Minor issues or recommendations

Line 30 – maybe change "are specific to distinct acyl-CoA substrates" to "have a distinct acyl-CoA substrate"
Line 31 – maybe instead of "unevenly occurring" change to "unevenly distributed".
Figure 1 – Title, change "AccA3/AccD4/AccD4/AccE" to "AccA3/AccD4/AccD5/AccE5"
Figure 1d – label stereochemistry inconsistency. (2S)-methylmalonyl-CoA?
Figure 1d – It is unclear what the top and bottom panels represent.
Line 49 – Wouldn't it be more appropriate to say this is pseudo 3-fold rotational symmetry with a true 2-fold symmetry

between the top and bottom of the CT rings?
Line 101 – change “AccA5” to “AccD5”
The methods citation 3 seems odd for the analysis of acyl-CoAs.

Signed: Jeremy Lohman, Michigan State University

Reviewer #2

(Remarks to the Author)
General remarks
The manuscript “Architecture of an asymmetric short chain/long chain hybrid acyl-CoA carboxylase” by Edukondalu Mullapudi, Hai Minh Thai, Luiz Pedro Sório de Carvalho, and Matthias Wilmanns describes the structural organization of the long-chain carboxylase (LCC) complex from Mycobacterium smegmatis. Using cryo-EM analysis of a purified complex, the authors have determined a novel architecture of this unusual ACCase assembly and provided structural and mechanistic insights into its previously described biochemical properties.
The work shows a highly asymmetric complex containing 16 subunits: a heterohexameric carboxyltransferase (CT) core, composed of two AccD4 and four AccD5 subunits, two AccA3 tetramers, each subunit containing by the biotin carboxylase and the biotin carboxyl domains (BC-BCCP), and two AccE5 ε-subunits. The Cryo-EM data provide important structural information about the role of AccE5 in anchoring the BC and CT assemblies and mediating conformational flexibility within the complex. The structural data also suggest that large conformational changes occur upon binding of different acyl-CoA substrates.
Overall, this is a technically strong and well-performed study that provides the first detailed structural view of the LCC complex and helps explain the biochemical singularities of this enzyme system. The cryo-EM data are of good quality and the structural analysis has provided valuable insights into the diverse architecture and function of mycobacterial ACCases. While the experimental work supports the conclusions and claims made by the authors, the manuscript would benefit from careful revision to improve clarity and precision. A careful editing of the text would significantly improve readability. Also, some statements regarding the novelty of the complex and the role of specific subunits should be considered or better supported by previous literature.

Specific comments
Abstract:
The abstract should be revised to better communicate the main findings and the experimental approach.
The term “endogenous extraction” is vague and should be replaced with a more precise description of the purification strategy.
In line 17 “short-chain (LC)” should be corrected to “short-chain (SC)”.
The statement that the presence of AccD4 and AccD5 is “triggered by a unique AccE5 dimer” is not well supported. The authors may want to indicate instead that AccE5 contributes to the stabilization or structural organization of the heterohexameric CT assembly.
In line 19 and throughout the manuscript, the authors refer to an “AccD5 dimer”, but the structural data do not demonstrate a defined dimeric unit. The terminology should therefore be reconsidered.

Main text:
Lines 31-35. The paragraph “Many mycobacteria contain an expanded repertoire of unevenly occurring ACCase genes, due to specific requirements for the biosynthesis of a complex cell envelope enriched with mycolic acids, which are critical for bacterial survival and pathogenicity. However, any mechanism by which ACCases with uneven stoichiometry assemble has remains unknown“ . Although the statement is correct, it sounds like you are trying to link the unevenly occurring ACCase genes with an uneven stoichiomestry, which may lead to a misinterpretation. The need of this genera of bacteria to produce a vast array of very complex lipids (not only mycolic acids) relates with the diversity of ACCases activities, and therefore, for the presence of a high number of genes encoding for their different subunits.

Line 38. The authors state that they “discovered a previously unknown ACCase complex.” However, an LCC enzyme complex composed of AccD4, AccD5, AccA3 and AccE5 has already been biochemically characterized from purified M. tuberculosis proteins, and demonstrated that this enzyme requires the four subunits to carboxylate both short- and long-chain acyl-CoA substrates (Bazet Lionnet et al, 2017, FEBS J, 284(7):1110-1125). The novelty of the present work lies mainly in the structural characterization and architectural organization of this complex rather than in the discovery of the enzyme activity itself. Mentioning the previous biochemical characterization of this system can naturally lead to its structural characterization as a way of understanding the molecular bases of its two distinctive enzyme activities.
Line 60-62. It is important to mention that homo-oligomeric CTs, like AccD5 in the ACCase 5 complex, also needs AccE5 to gain full activitiy (Gago, et al, J of Bacteriology, 2006, p. 477–486; Oh, et al, 2006, J Biol Chem 281:3899–3908), suggesting that this small subunit may play similar role in this complex than in the LCC complex. It might be relevant to discuss this observation.
Lines 70–74. This sentence should be rewritten for clarity.
Line 75. The phrase “this barrel” should specify which structural barrel is being referred to.
Line 88. Replace “AccD4 and AccD5” with “AccD4 or AccD5” if appropriate.
Line 89. The stoichiometry may be more clearly expressed as $\alpha 8:\beta 6$.
Figures:
Figure 1. The description of the peaks in panel 1d should be clarified. In particular, the text should explain more clearly the presence or absence of specific peaks and their relation to the different enzyme activities.

In addition, a clearer description of the LCC enzymatic assay methodology should be provided, including which purification fractions were used for the activity measurements.
Figure 2. In the title and legend, the complex should be described as A3/D4/D5/E5.In panel d, “N256(D3)” should be corrected to “N256(A3)”.
Extended data:
Extended Data Figure 1. The rationale for panel c should be clarified. It is not clear why this elution profile and gel are presented, particularly since they include the AccD1 protein.
In addition, AccE5 appears to migrate anomalously considering its predicted molecular weight (~10–12 kDa). A brief comment explaining this observation would be helpful.

Final recommendation
In summary, this manuscript provides important structural insight into the architecture of the LCC acomplex from Mycobacterium smegmatis. The cryo-EM analysis offers the first detailed structural view of this hybrid ACCase assembly and contributes to understanding the organization and function of mycobacterial acyl-CoA carboxylases.
Overall, I believe that the manuscript will be suitable for publication after the authors address the comments and suggestions outlined above.

Reviewer #3

(Remarks to the Author)
This paper describes the structure of a mycobacterial hybrid asymmetric acyl-CoA-carboxylase (ACCase) complex. All the structures of ACCase’s to date are those of symmetrical complexes that are specific to distinct acyl-CoA substrates.

Due to the complexity of the mycobacterial cell-wall containing a variety of fatty acids and lipids, mycobacteria have a vast array of ACCase genes, and many of the clusters have an odd number of genes, resulting in asymmetric AACases. To address the unknown mechanism of assembly of these asymmetric ACCases, the authors purified endogenous ACCases from Mycobacterium smegmatis and discovered a unique ACCase that had two different carboxyl transferase (CT) subunits, suggesting a hybrid asymmetric ACCase along with an additionally AccE5 subunit. The authors showed that the complex had activity against short chained and long chained CoAs, confirming its hybrid nature. By cryo-electron microscopy (cryo-EM), the authors solved the structures of the hybrid ACCase complex both in the absence and presence of long chain and short chain acyl-CoA substrates. The architecture is 16-subunits with (AccD4)2/(AccD5)4 hexamer in the center forming a double-ring (barrel-like) arrangement and linked to 2x(AccD3)4 at the top and base of the ring arrangement by the two AccE5 linker subunits that form a central axis of the complex.

In its apo form, the ACCase appears to have a near 2-fold symmetry through the plane between the double ring arrangement; however the top (AccD3)4 arrangement tilts 11 degrees from the AccE5 dimer central axis due to the uneven arrangement of the (AccD4)2/(AccD5)4 hexamer. Notably, when acyl-CoA is bound, this asymmetry is further enhanced in both (AccD3)4 tetramer arrangements by tilting 14 degrees from the AccE5 dimer central axis together with a ~90 degrees rotation around the barrel segment. This results in some of the AccD3 subunits to tilt near or contacting the AccD4(5) subunits of the barrel.

This conformational change highlights a mechanism of action, where binding of the long chain or short chain CoAs to the AccD4(5) subunits, respectively, results in a major conformation change where the AccD3 subunit maybe transfer its carboxyl biotin to the AccD4(5) active sites. Finally, the authors describe how each AccD4 subunit requires two AccA3 subunits to mediate delivery of carboxy biotin, which the AccD5 subunit requires only a single AccA3 subunit. The authors also describe structural features which facilitates this flexibility and also the complex’s multi-substrate capacity.

This manuscript describes the first structure of a hybrid asymmetric ACCase complex and sheds light on its mechanism of action by solved the structures of catalytically active endogenously purified mycobacterial multi-subunit complexes in the absence and presence of long chain and short chain CoAs. Three structures are solved to high resolution by cryo-EM. The manuscript is well written, and the two figures have the required detail and labeling to allow the reader to visualize the most important features of the structures along with the mechanism of action.

The only question I have is ‘what is the specificity of the hybrid ACCase to the long-chain and short-chain CoAs in mycobacetria’ but this is only a minor comment.

Version 1:

Reviewer comments:

Reviewer #1

(Remarks to the Author)
The reviewers have adequately addressed all of my concerns.

Reviewer #2

(Remarks to the Author)
The authors have adequately addressed all the concerns raised in my previous review. They have improved the clarity of the manuscript and incorporated the requested additions. I consider the revised version suitable for publication.

Open Access This Peer Review File is licensed under a Creative Commons Attribution 4.0 International License, which permits use, sharing, adaptation, distribution and reproduction in any medium or format, as long as you give appropriate credit to the original author(s) and the source, provide a link to the Creative Commons license, and indicate if changes were made.
In cases where reviewers are anonymous, credit should be given to 'Anonymous Referee' and the source.
The images or other third party material in this Peer Review File are included in the article's Creative Commons license, unless indicated otherwise in a credit line to the material. If material is not included in the article's Creative Commons license and your intended use is not permitted by statutory regulation or exceeds the permitted use, you will need to obtain permission directly from the copyright holder.
To view a copy of this license, visit https://creativecommons.org/licenses/by/4.0/

The author's responses are in blue. Changes in the text of the manuscript are also highlighted in blue.

Referee expertise:

Referee #1: Structural Biology and microbial biochemistry

Referee #2: Physiology and biochemistry of Tuberculosis

Referee #3: structural biology of microbial enzymes

Reviewers' comments:

Reviewer #1 (Remarks to the Author):

The article "Architecture of an asymmetric short chain/long chain hybrid acyl-CoA carboxylase" is a unique and important addition to the field of carboxyltransferase structure-function. The authors determine structures of a Mycobacterial acyl-CoA carboxylase enzyme complex. This complex is unique compared to ones previously solved due to asymmetry in three senses, i) asymmetry in the typically homohexameric carboxyltransferase (CT) core whereby three short-chain carboxyltransferase dimers have one dimer replaced by a long-chain carboxyltransferase dimer, ii) the trimeric biotin-carboxylase (BC)/biotin carboxy carrier protein (BCCP) is replaced by tetrameric versions, iii) a common 8-stranded beta-barrel with a central alpha-helix that interfaces between the BC and CT is truncated to two beta-sheets from each BC/BCCP but includes a peptide that comprises the central alpha-helix and contributes one strand to the beta-barrel. The arrangement leads to an exaggerated asymmetry. This structure helps resolve a mystery for why there are multiple carboxyltransferase subunits in Mycobacteria. This structure also raises many questions beyond the mycobacterial system. In other Actinobacteria, there appear to be orphan carboxyltransferase domains, could this AccA3/D4/D5/E5 asymmetric system in fact represent a majority of Actinobacterial carboxyltransferase systems? Has the field been missing these asymmetric complexes due to an insistence of using heterologous expression? While this manuscript does not directly address these questions, it will inspire the field to reassess the study of carboxyltransferase complexes using native expression.

I don't see any major issues with the data collection or processing statistics and the authors don't appear to be overinterpreting their data. The most important questions being addressed are the stoichiometry and overall composition of the complex, which is interpretable from the multiple data sets collected. A more thorough characterization of the complex would make the paper more satisfying, such as, what are the relative carboxylation rates for SC vs LC substrates? However, such experiments are not needed to justify the reported structures.

For reasons of time and capacity we were unable to conduct more in-depth biochemical experiments for this brief communication.

I only have questions that the authors could speculate on for why such a complex might exist.

Does the ratio of AccD4 to AccD5 in the complex suggest that the enzyme is balancing the production of SC and LC malonyl-CoAs towards the SCs?

For such evaluation, there would be a requirement for relevant experiments *in vivo*. For reasons of time and capacity we unable to conduct more in-depth biochemical experiments for this brief communication.

Do the authors think there could be shuttling of carboxyl-groups between the SC and LC active sites? Could this be a role for the “extra” BC/BCCPs in the complex?

Investigating trajectories of shuttling pathways and stoichiometry is one of the key questions, arising from the unexpected arrangement found in this ACCase complex. For reasons of time and capacity we unable to conduct more in-depth biochemical experiments for this brief communication.

Due to errors in the .pdf provided the methods for Bacterial strain and growth conditions and Mass spectrometry are missing and can’t be evaluated.

We apologize for this. The revised version of the manuscript includes all relevant sections.

Minor issues or recommendations

Line 30 – maybe change “are specific to distinct acyl-CoA substrates” to “have a distinct acyl-CoA substrate”

We have revised the text accordingly.

Line 31 – maybe instead of “unevenly occurring” change to “unevenly distributed”.

We are concerned that using the term “distributed” could create an impression on an uneven distribution with respect to the overall operon organization. What we intend to refer to is an uneven number of genes throughout the entire genome, regardless of their allocation to specific operons. Therefore, we have kept the term “occurring”, but we are open to any changes that may express this idea more clearly.

Figure 1 – Title, change “AccA3/AccD4/AccD4/AccE” to “AccA3/AccD4/AccD5/AccE5”

The title of Figure 1 has been corrected accordingly.

Figure 1d – label stereochemistry inconsistency. (2S)-methylmalonyl-CoA?

For the sake of consistency, we have removed the stereochemistry specifications in the revised Figure (now Figure 1e).

Figure 1d – It is unclear what the top and bottom panels represent.

We have revised the figure (previously Figure 1d) to improve its clarity (Figure inserted). Since the revised version requires more space, we have separated it from the structural content of the previous Figure 1 (now Figure 1e) and have combined into with the content of the previous Extended Data Figure 1 (now Figure 1a-d).

Figure 1

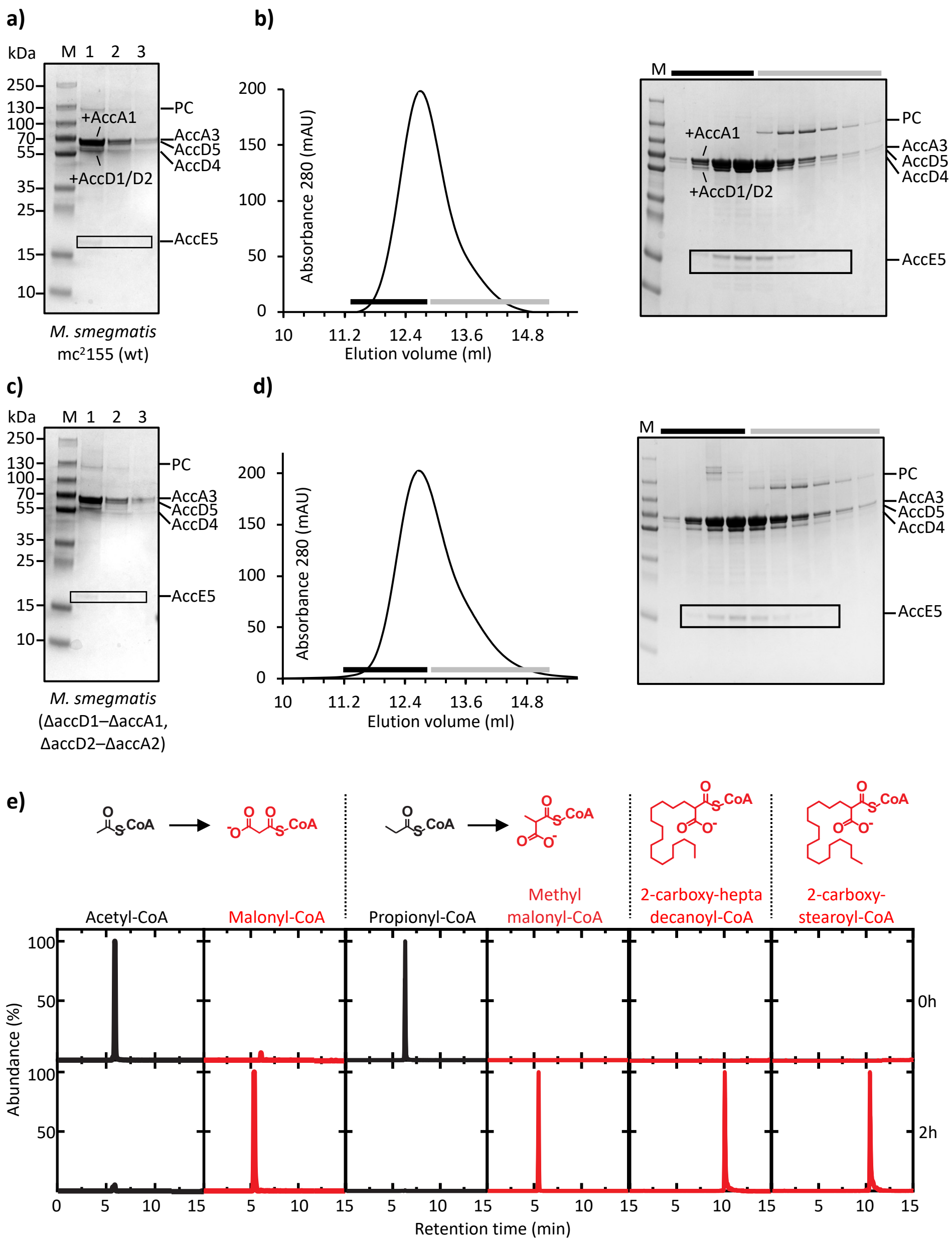

We have performed the following changes:

Figure 1a, c: the pull down data for both the *M. smegmatis* mc^{2}155 (panel a) and the *M. smegmatis* (Δ*accD1*-Δ*accA1*, Δ*accD2*-Δ*accA2*) (panel c) strains are shown.

Figure 1b, d: we have changed the colors of the bars indicating the fractions used for further characterization from green/blue to black/gray to avoid confusion with the colors used in the structural presentations.

Figure 1e: In the upper part, the four substrates (black) /products (red) assayed are indicated by their chemical structures and names. The four assays are separated by vertical dashed lines. For experiments, where only products were detectable, no substrate panels are shown. The ion counts shown for substrates and products are overlays from threefold repeated experiments. The complete data from these experiments are in the source data file. Since the ion count statistics for each substrate and product are different, they cannot be directly compared. Therefore, we now show the ion counts on a relative scale, where 100% is equivalent to the maximum count observed in each experiment.

The captions of all panels have been substantially expanded. All changes are highlighted in blue.

Line 49 – Wouldn't it be more appropriate to say this is pseudo 3-fold rotational symmetry with a true 2-fold symmetry between the top and bottom of the CT rings?

The measured angle between neighboring AccD4 and AccD5 subunits is 120.05 degrees. Taking estimated measurement errors into account, in our view it is legitimate to categorize this as molecular 3-fold symmetry. One could argue that the symmetry relations between different subunit identities can only be a pseudo-symmetric by definition. As we observe substantial symmetry deviations in other parts of the holo complex, it may be advantageous to clarify what – at the level of available structural resolution and precision – appears to be symmetric and what clearly deviates from symmetry, allowing to classify the latter properties as "asymmetric". That said, we are open to adopting any further changes that would be beneficial for the manuscript.

Line 101 – change "AccA5" to "AccD5"

We have revised the text accordingly.

The methods citation 3 seems odd for the analysis of acyl-CoAs.

The citation has been removed. Since the procedure described is an author's laboratory protocol, we made sure that it is written in sufficient detail to allow for its reproduction. The text has been reviewed and a few minor modifications have been applied. These modifications are highlighted in blue color.

Signed: Jeremy Lohman, Michigan State University

Reviewer #2 (Remarks to the Author):

General remarks

The manuscript "Architecture of an asymmetric short chain/long chain hybrid acyl-CoA carboxylase" by Edukondalu Mullapudi, Hai Minh Thai, Luiz Pedro Sório de Carvalho, and Matthias Wilmanns describes the structural organization of the long-chain carboxylase (LCC) complex from Mycobacterium smegmatis. Using cryo-EM analysis of a purified complex, the authors have determined a novel architecture of this unusual ACCase assembly and provided structural and mechanistic insights into its previously described biochemical properties.

The work shows a highly asymmetric complex containing 16 subunits: a heterohexameric carboxyltransferase (CT) core, composed of two AccD4 and four AccD5 subunits, two AccA3 tetramers, each subunit containing by the biotin carboxylase and the biotin carboxyl domains (BC-BCCP), and two AccE5 ε-subunits. The Cryo-EM data provide important structural information about the role of AccE5 in anchoring the BC and CT assemblies and mediating conformational flexibility within the complex. The structural data also suggest that large conformational changes occur upon binding of different acyl-CoA substrates.

Overall, this is a technically strong and well-performed study that provides the first detailed structural view of the LCC complex and helps explain the biochemical singularities of this enzyme system. The cryo-EM data are of good quality and the structural analysis has provided valuable insights into the diverse architecture and function of mycobacterial ACCases.

While the experimental work supports the conclusions and claims made by the authors, the manuscript would benefit from careful revision to improve clarity and precision. A careful editing of the text would significantly improve readability. Also, some statements regarding the novelty of the complex and the role of specific subunits should be considered or better supported by previous literature.

Specific comments

Abstract:

The abstract should be revised to better communicate the main findings and the experimental approach.

We have reformulated and expanded the abstract accordingly, keeping to a limit of 150 words.

The term "endogenous extraction" is vague and should be replaced with a more precise description of the purification strategy.

In the revised version, we are using the formulation "..we pulled down endogenously expressed biotinylated proteins from *Mycobacterium smegmatis…"*

In line 17 "short-chain (LC)" should be corrected to "short-chain (SC)".

We have revised the text accordingly.

The statement that the presence of AccD4 and AccD5 is “triggered by a unique AccE5 dimer” is not well supported. The authors may want to indicate instead that AccE5 contributes to the stabilization or structural organization of the heterohexameric CT assembly.

We have replaced “triggered” by “established”.

We would like to emphasize that there are distinct sets of specific interactions between the two protomeric units of the end-to-end C-terminal AccE5 dimer with the single AccD4 subunit and the two AccD5 subunits within each layer of the central hexameric CT assembly. For further details, see especially Figure 3a.

In line 19 and throughout the manuscript, the authors refer to an “AccD5 dimer”, but the structural data do not demonstrate a defined dimeric unit. The terminology should therefore be reconsidered.

As mentioned above and illustrated in Figure 3c, the two AccD5 subunits actually do form an end-to-end C-terminal dimer with twofold internal symmetry, which is connected by a set of specific interactions. The twofold axis that defines the relation of the two AccD5 protomers is also indicated in Figure 3c.

Main text:

Lines 31-35. The paragraph “Many mycobacteria contain an expanded repertoire of unevenly occurring ACCase genes, due to specific requirements for the biosynthesis of a complex cell envelope enriched with mycolic acids, which are critical for bacterial survival and pathogenicity. However, any mechanism by which ACCases with uneven stoichiometry assemble has remains unknown“ . Although the statement is correct, it sounds like you are trying to link the unevenly occurring ACCase genes with an uneven stoichiomestry, which may lead to a misinterpretation. The need of this genera of bacteria to produce a vast array of very complex lipids (not only mycolic acids) relates with the diversity of ACCases activities, and therefore, for the presence of a high number of genes encoding for their different subunits.

We have reformulated the sentence in the following way: “However, the overall composition of these ACCase genes into stoichiometric holo complexes remains unknown.”

Line 38. The authors state that they “discovered a previously unknown ACCase complex.” However, an LCC enzyme complex composed of AccD4, AccD5, AccA3 and AccE5 has already been biochemically characterized from purified M. tuberculosis proteins, and demonstrated that this enzyme requires the four subunits to carboxylate both short- and long-chain acyl-CoA substrates (Bazet Lionnet et al, 2017, FEBS J, 284(7):1110-1125).

We thank the referee for this comment.

We have added the following to the preceding paragraph: “Albeit the existence of mycobacterial ACCase complexes with multiple substrate specificities was proposed previously (8), the overall composition of these ACCase genes in stoichiometric holo complexes has remained unknown.” We reformulated the disputed sentence as follows: ”We identified and characterized an endogenously expressed hybrid ACCase complex, which is composed of one distinct BC subunit (AccA3), two different CT subunits (AccD4, AccD5) and a unique ε-subunit (AccE5) subunit (Figure 1a,b, Supplementary Table 1).”

The novelty of the present work lies mainly in the structural characterization and architectural organization of this complex rather than in the discovery of the enzyme activity itself. Mentioning the previous biochemical characterization of this system can naturally lead to its structural characterization as a way of understanding the molecular bases of its two distinctive enzyme activities.

See response above.

Line 60-62. It is important to mention that homo-oligomeric CTs, like AccD5 in the ACCase 5 complex, also needs AccE5 to gain full activitiy (Gago, et al, J of Bacteriology, 2006, p. 477–486; Oh, et al, 2006, J Biol Chem 281:3899–3908), suggesting that this small subunit may play similar role in this complex than in the LCC complex. It might be relevant to discuss this observation.

We have added the following: “Furthermore, although a functional role of the ε-subunit AccE5 in promoting catalytic AccD5 CT activity was established (9, 10), any mechanistic insight into its contribution to the corresponding holo complex remained elusive.”

Lines 70–74. This sentence should be rewritten for clarity.

We have modified the sentence as follows: “Although similar β-barrel motifs are found in single BC subunits of canonical single-substrate ACCase complexes as well (1, 6), in the structure of this hybrid SC/LC ACCase complex the barrel differs fundamentally due to the contribution of all four AccA3 subunits and the insertion of a single AccE5 β-strand, thus generating an asymmetric arrangement.”

Line 75. The phrase “this barrel” should specify which structural barrel is being referred to.

We have replaced “this barrel” by “the $(AccA3)_4$/AccE5 barrel”.

Line 88. Replace “AccD4 and AccD5” with “AccD4 or AccD5” if appropriate.

We have revised the text accordingly.

Line 89. The stoichiometry may be more clearly expressed as α8:β6.

We have replaced the phrase in the following: "However, the resulting 8:6 stoichiometry of 2 x 4 AccA3 and 6 AccD4/AccD5 subunits". We prefer this formulation, as "stoichiometry" is generally expressed in numbers only, referring to specific entities.

Figures:
Figure 1. The description of the peaks in panel 1d should be clarified. In particular, the text should explain more clearly the presence or absence of specific peaks and their relation to the different enzyme activities.

See reply to equivalent comment by referee #1.

In addition, a clearer description of the LCC enzymatic assay methodology should be provided, including which purification fractions were used for the activity measurements.

In the caption of Figure 1b, d we have added the following: "b, d size-exclusion chromatography profiles of the purified AccA3/AccD4/AccD5/AccE5 holo complexes and SDS–PAGE of fractions across the main elution peak. Fractions, used for biochemical and structural analysis, are marked by a black bar; fractions that also containing pyruvate carboxylase are marked by a grey bar and have been discarded from further characterization."

In the section "Protein Purification" we have added the following: "Fractions containing the purified AccA3/AccD4/AccD5/AccE5 holo complex were pooled and concentrated using a centrifugal filter unit **(Figure 1b, d)**. The fractions indicated by black bars from *M. smegmatis* mc^2155 strain were used for apo cryo-EM structure determination**.** The fractions from the *M. smegmatis* (Δ*accD1*-Δ*accA1*, Δ*accD2*-Δ*accA2*) strain were used for activity assays and cryo-EM studies of substrate-bound complexes."

For further details on enzymatic assays see reply to referee #1.

Figure 2. In the title and legend, the complex should be described as A3/D4/D5/E5.

We have added the following to the caption of Figure 2: "Acronym shortcuts used: AccA3, A3; AccD4, D4; AccD5, D5, AccE5, E5". This information is also included in the Figure 1 caption, but it could be covered by the statement in the Figure 2 caption "All other conventions are as in **Figure 1."** Please note that the motivation to use these acronym shortcuts was to reduce the text load in both figures. Where there is sufficient space, we use the correct acronyms (AccA3, AccD4, AccD5, and AccE5) throughout the text and in the figures.

In panel d, "N256(D3)" should be corrected to "N256(A3)".

We have corrected the label to N256(D4).

Extended data:

Extended Data Figure 1. The rationale for panel c should be clarified. It is not clear why this elution profile and gel are presented, particularly since they include the AccD1 protein.

As pointed out above, this figure has now been merged with the biochemical assays data (previously Figure 1d) into a revised Figure 1. For the sake of clarity, we now show the protein purification data from extracts of both *M. smegmatis mc2155* (panels a, b)and *M. smegmatis* (ΔaccD1-ΔaccA1, ΔaccD2-ΔaccA2) (panels c, d) strains.

For biochemical characterization and the structures in the presence of acyl-CoA substrate, we used samples from the *M. smegmatis* (ΔaccD1-ΔaccA1, ΔaccD2-ΔaccA2) strain, to avoid any contamination with AccD1/AccA1 holo complex.

For further details, see response to reviewer 1.

In addition, AccE5 appears to migrate anomalously considering its predicted molecular weight (~10–12 kDa). A brief comment explaining this observation would be helpful.

We have added the following: "The estimated molecular weights are in agreement with the mass spectrometry data, except for AccE5, which appeared at a higher apparent molecular weight on SDS–PAGE, probably due to its partly intrinsically unfolded nature **(Supplementary Table 1)."**

Final recommendation
In summary, this manuscript provides important structural insight into the architecture of the LCC acomplex from Mycobacterium smegmatis. The cryo-EM analysis offers the first detailed structural view of this hybrid ACCase assembly and contributes to understanding the organization and function of mycobacterial acyl-CoA carboxylases.
Overall, I believe that the manuscript will be suitable for publication after the authors address the comments and suggestions outlined above.

Reviewer #3 (Remarks to the Author):

This paper describes the structure of a mycobacterial hybrid asymmetric acyl-CoA-carboxylase (ACCase) complex. All the structures of ACCase's to date are those of symmetrical complexes that are specific to distinct acyl-CoA substrates.

Due to the complexity of the mycobacterial cell-wall containing a variety of fatty acids and lipids, mycobacteria have a vast array of ACCase genes, and many of the clusters have an odd number of genes, resulting in asymmetric AACases. To address the unknown mechanism of assembly of these asymmetric ACCases, the authors purified endogenous ACCases from Mycobacterium smegmatis and discovered a unique ACCase that had two different carboxyl transferase (CT) subunits, suggesting a hybrid asymmetric ACCase along with an additionally AccE5 subunit. The authors showed that the complex had activity against short chained and long chained CoAs, confirming its hybrid nature. By cryo-electron microscopy (cryo-EM), the

authors solved the structures of the hybrid ACCase complex both in the absence and presence of long chain and short chain acyl-CoA substrates. The architecture is 16-subunits with (AccD4)2/(AccD5)4 hexamer in the center forming a double-ring (barrel-like) arrangement and linked to 2x(AccD3)4 at the top and base of the ring arrangement by the two AccE5 linker subunits that form a central axis of the complex.

In its apo form, the ACCase appears to have a near 2-fold symmetry through the plane between the double ring arrangement; however the top (AccD3)4 arrangement tilts 11 degrees from the AccE5 dimer central axis due to the uneven arrangement of the (AccD4)2/(AccD5)4 hexamer. Notably, when acyl-CoA is bound, this asymmetry is further enhanced in both (AccD3)4 tetramer arrangements by tilting 14 degrees from the AccE5 dimer central axis together with a ~90 degrees rotation around the barrel segment. This results in some of the AccD3 subunits to tilt near or contacting the AccD4(5) subunits of the barrel.

This conformational change highlights a mechanism of action, where binding of the long chain or short chain CoAs to the AccD4(5) subunits, respectively, results in a major conformation change where the AccD3 subunit maybe transfer its carboxyl biotin to the AccD4(5) active sites. Finally, the authors describe how each AccD4 subunit requires two AccA3 subunits to mediate delivery of carboxy biotin, which the AccD5 subunit requires only a single AccA3 subunit. The authors also describe structural features which facilitates this flexibility and also the complex's multi-substrate capacity.

This manuscript describes the first structure of a hybrid asymmetric ACCase complex and sheds light on its mechanism of action by solved the structures of catalytically active endogenously purified mycobacterial multi-subunit complexes in the absence and presence of long chain and short chain CoAs. Three structures are solved to high resolution by cryo-EM. The manuscript is well written, and the two figures have the required detail and labeling to allow the reader to visualize the most important features of the structures along with the mechanism of action.

The only question I have is 'what is the specificity of the hybrid ACCase to the long-chain and short-chain CoAs in mycobacetria' but this is only a minor comment.

Our current knowledge on specificity is described by the assay data in Figure 1c and associated text.
