# Supplementary material for: Architecture of an asymmetric short chain/long chain hybrid acyl‑CoA carboxylase from Mycobacterium smegmatis
Source: Commun Biol. 2026 Jun 11;9:1251. doi: 10.1038/s42003-026-10439-x (PMC13612425; doi:10.1038/s42003-026-10439-x)
Supplement: Supplementary file 2 [file 42003_2026_10439_MOESM2_ESM.pdf]

Supplementary Table 1. LC–MS/MS identification of ACCase subunits

Protein identified	Molecular weight (kDa)	Total intensity	Max HyperScore	Coverage (%)	Total intensity	Max HyperScore	Coverage (%)
		M. smegmatis mc^{2}155			*M. smegmatis* Δ*accD1*-Δ*accA1*, Δ*accD2*-Δ*accA2*		
AccA3	63.1	4.59E+10	85.5	91.0	3.22E+10	83.9	78.3
AccD5	58.4	9.35E+10	79.7	71.8	7.29E+10	71.3	71.0
AccD4	56.2	5.73E+10	82.2	83.4	5.07E+09	73.0	82.0
AccE5	10.3	2.03E+09	30.5	63.8	1.09E+09	37.8	63.8
AccA1	70.0	6.28E+05	—	—	—	—	—
AccD1	54.8	2.37E+08	66.5	57.7	—	—	—
AccD2	56.6	3.95E+07	56.4	37.1	—	—	—

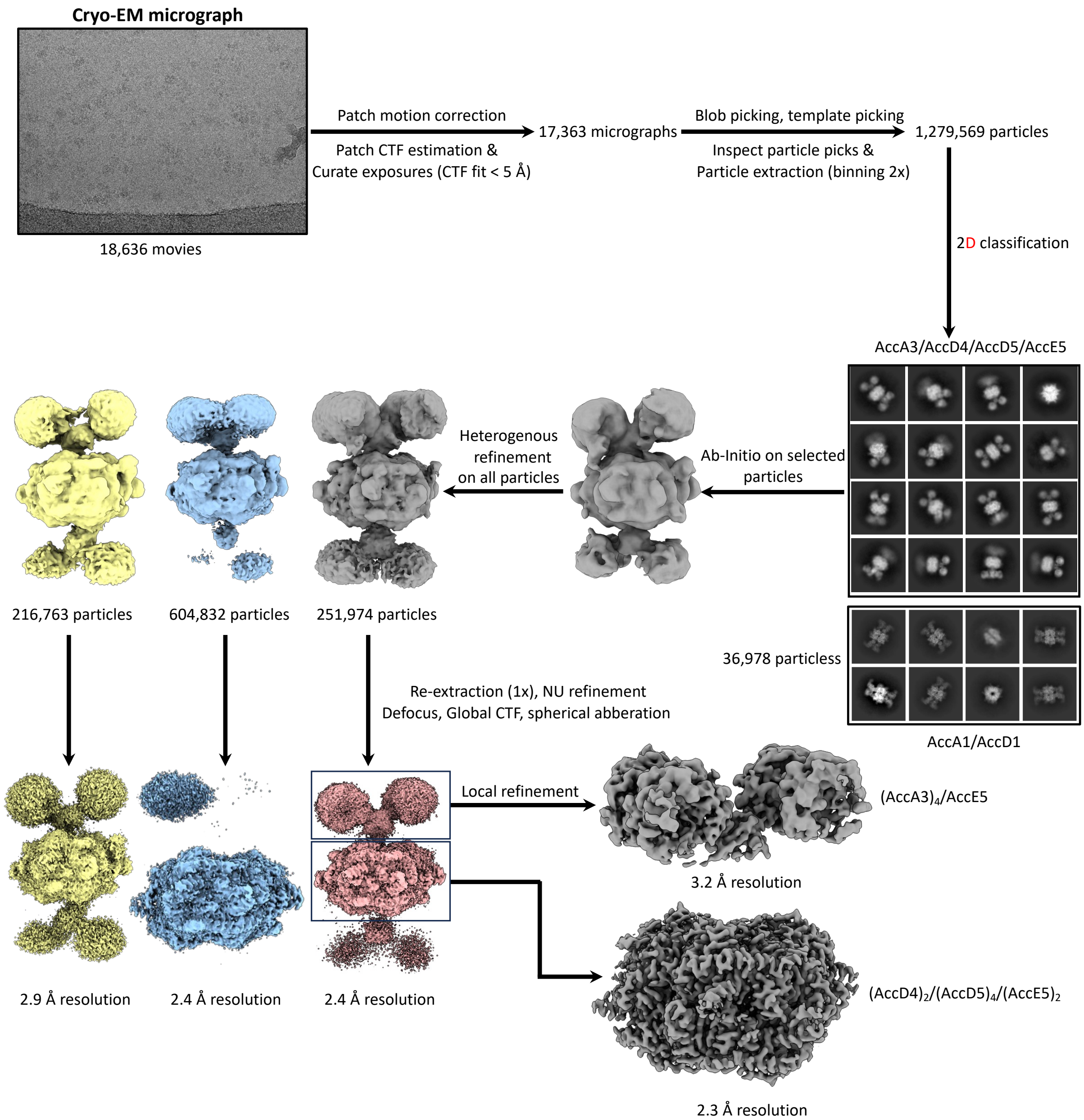

Supplementary Figure 1: Cryo-EM data processing workflow for AccA3/AccD4/AccD5/AccE5 holo complex. Schematic of the cryo-EM data processing pipeline, performed in cryoSPARC. A total of 18,636 movies was processed, yielding 1,279,569 particles. After 2D classification and several rounds of *ab initio* and heterogeneous refinement, a subset of 251,974 particles was selected. This particle set was subjected to re-extraction and non-uniform (NU) refinement,

resulting in a 2.4 Å resolution map. Subsequent local refinement resolved the $(AccD4)_2/(AccD5)_4/(AccE5)_2$ core to 2.3 Å resolution and one of the two $(AccA3)_2$/AccE5 segments to 3.2 Å resolution. Composite maps were subsequently generated in ChimeraX for model building and refinement.

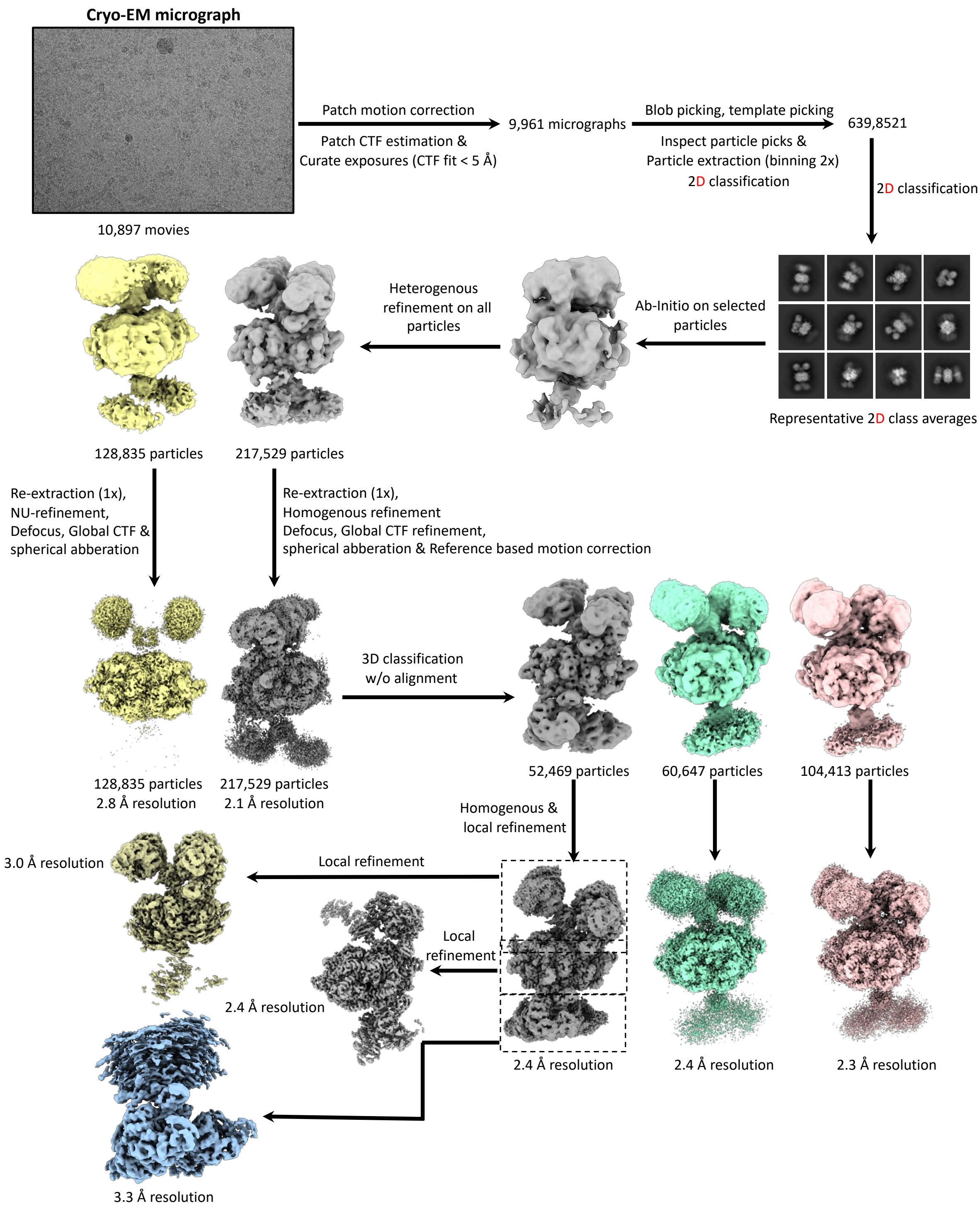

Supplementary Figure 2: Cryo-EM data processing workflow for AccA3/AccD4/AccD5/AccE5 complex with propionyl-CoA. A dataset of 10,897 movies was processed. Several rounds of 2D-classification, *ab initio* and heterogeneous refinement were performed to obtain a homogenous set of particles. Two classes corresponding to particle sets of 128,835 and 217,529 were selected for homogenous refinement, which yielded resolutions of 2.8 and 2.1 Å, respectively. Maps

corresponding to 217,529 particles were sorted using 3D classification without alignment. This sorting resulted in multiple subsets of 52,469, 60,647 and 104,413 particles, which were then subjected to homogenous refinement that yielded maps with resolutions between 2.3 and 2.4 Å . The class that displayed the complete complex was used for local refinements focusing on the upper $(AccA3)_2$/AccE5 region, central $(AccD4)_2$/$(AccD5)_4$/$(AccE5)_2$ core and the bottom $(AccA3)_2$/AccE5 region that yielded resolutions of 3.0, 2.4 and 3.3 Å, repectively.

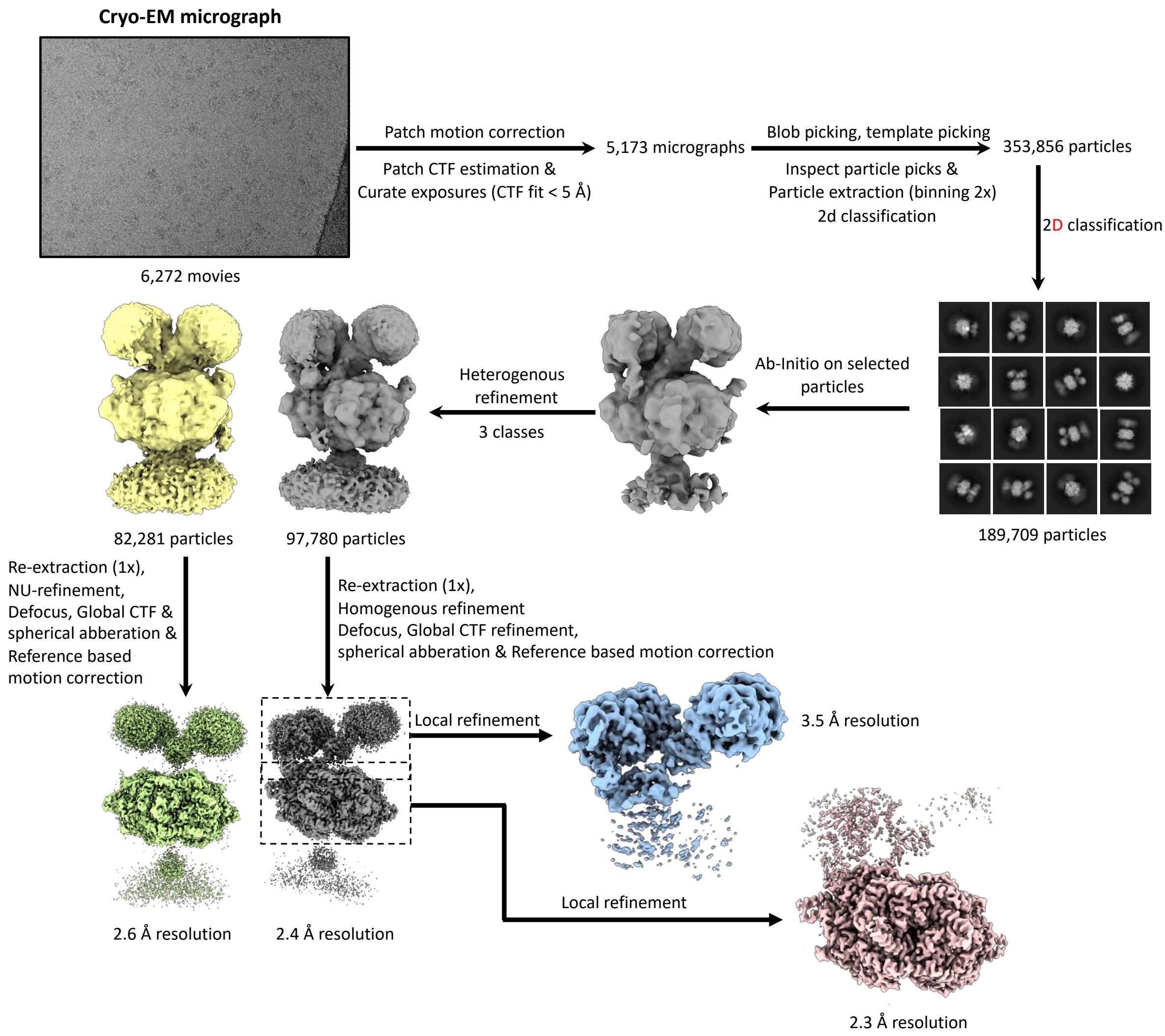

Supplementary Figure 3: Cryo-EM data processing workflow for AccA3/AccD4/AccD5/AccE5 complex with Arachidyl-CoA. From 6,272 movies, an initial set of 353,856 particles was picked. After 2D classification, 189,709 particles were used for heterogeneous refinement to generate three classes. The resulting particle subsets 82,281 and 97,780 particles were further refined yielding 2.6 Å and 2.4 Å maps. The class with 82, 281 particels was used for local refinement one of the two $(AccA3)_2$/AccE5 region and central $(AccD4)_2/(AccD5)_4/(AccE5)_2$ core of the complex, yielding final maps at 3.5 Å and 2.4 Å resolution.

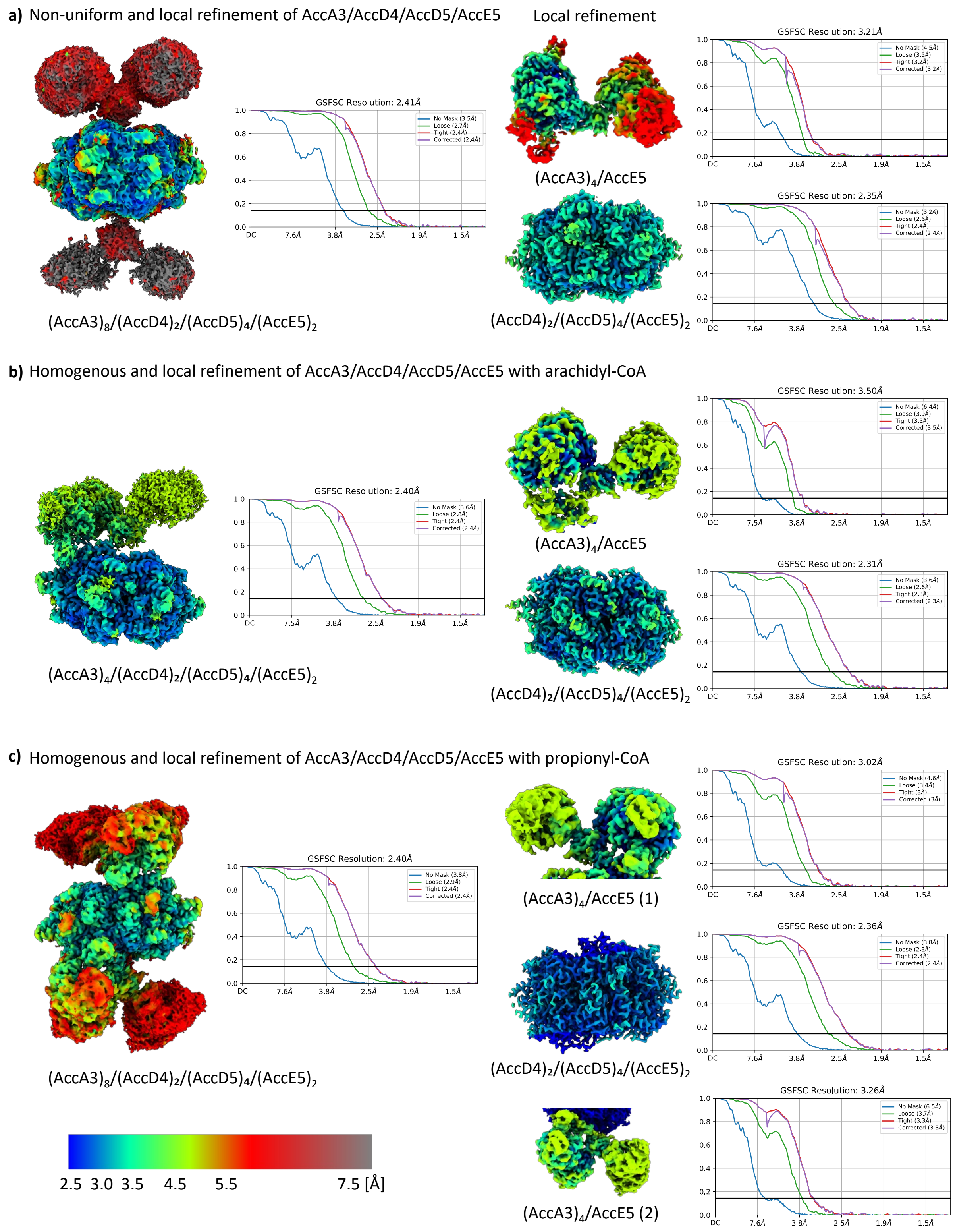

Supplementary Figure 4: Local resolution estimation of AccA3/AccD4/AccD5/AccE5 complex without and with acyl-CoA substrates. a, non-uniform refinement of AccA3/AccD4/AccD5/AccE5 holo complex without acyl-CoA substrates. The overall map is at

resolution 2.4 Å. Subsequent local refinement of this map resolved one of the two $(AccA3)_2$/AccE5 region to 3.2 Å and the $(AccD4)_2/(AccD5)_4/(AccE5)_2$ core to 2.4 Å resolution. **b,** homogenous refinement of AccA3/AccD4/AccD5/AccE5 in the presence of arachidyl-CoA. The overall map is at 2.4 Å resolution. Local refinement resolved the upper region to 3.5 Å and the core to 2.3 Å resolution. **c,** homogenous refinement of AccA3/AccD4/AccD5/AccE5 with propionyl-CoA: The overall map is at 2.4 Å resolution. Further 3D classification and local refinement yielded maps with resolutions of 3.0 Å , 2.4 Å , and 3.3 Å. The maps are colored according to local resolution (in Å), as indicated by a color bar. The GSFSC plots show the correlation between two independent half-maps, with the final resolution determined by the 0.143 FSC criterion (horizontal black line).

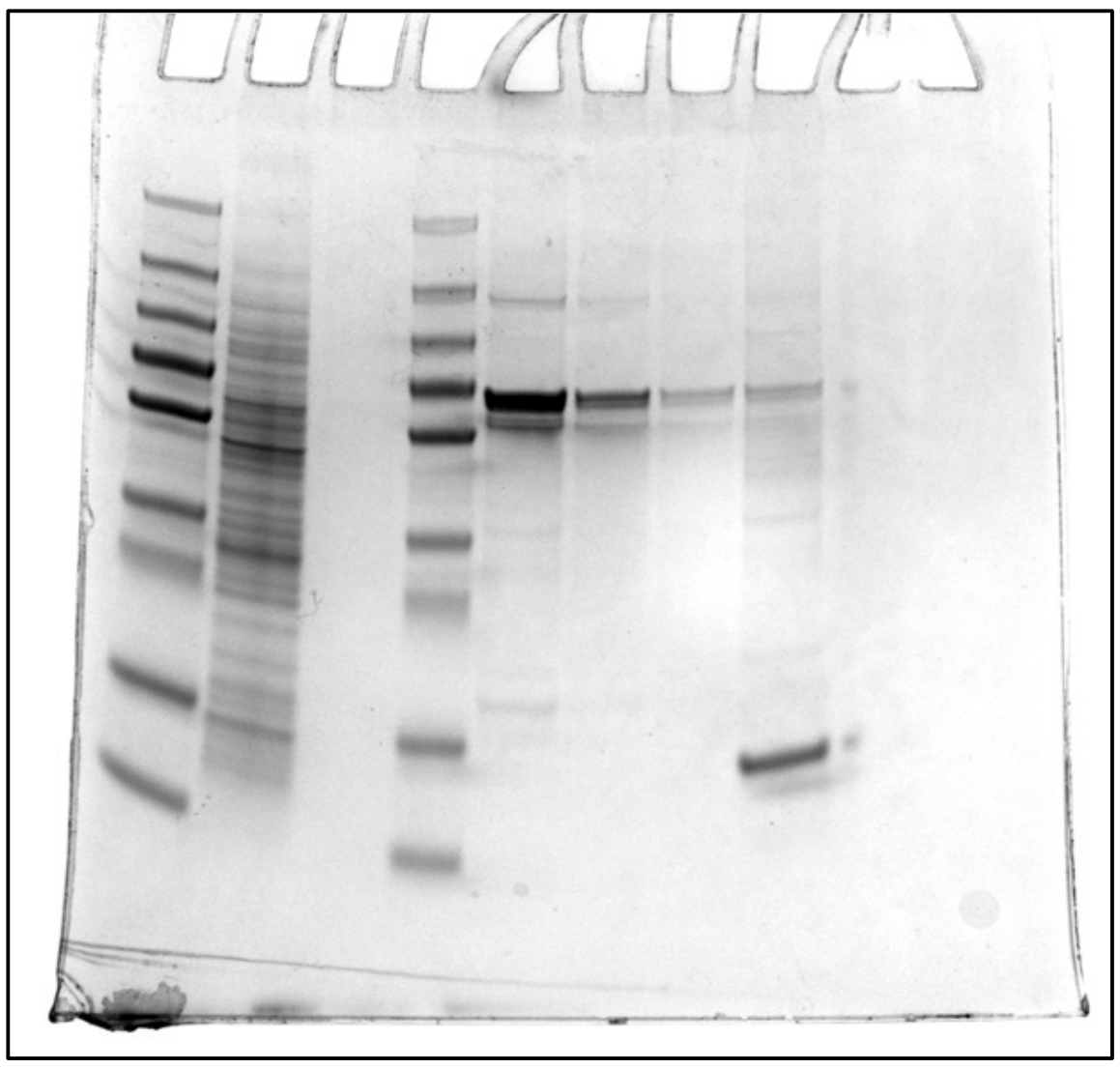

Fig. 1a uncropped

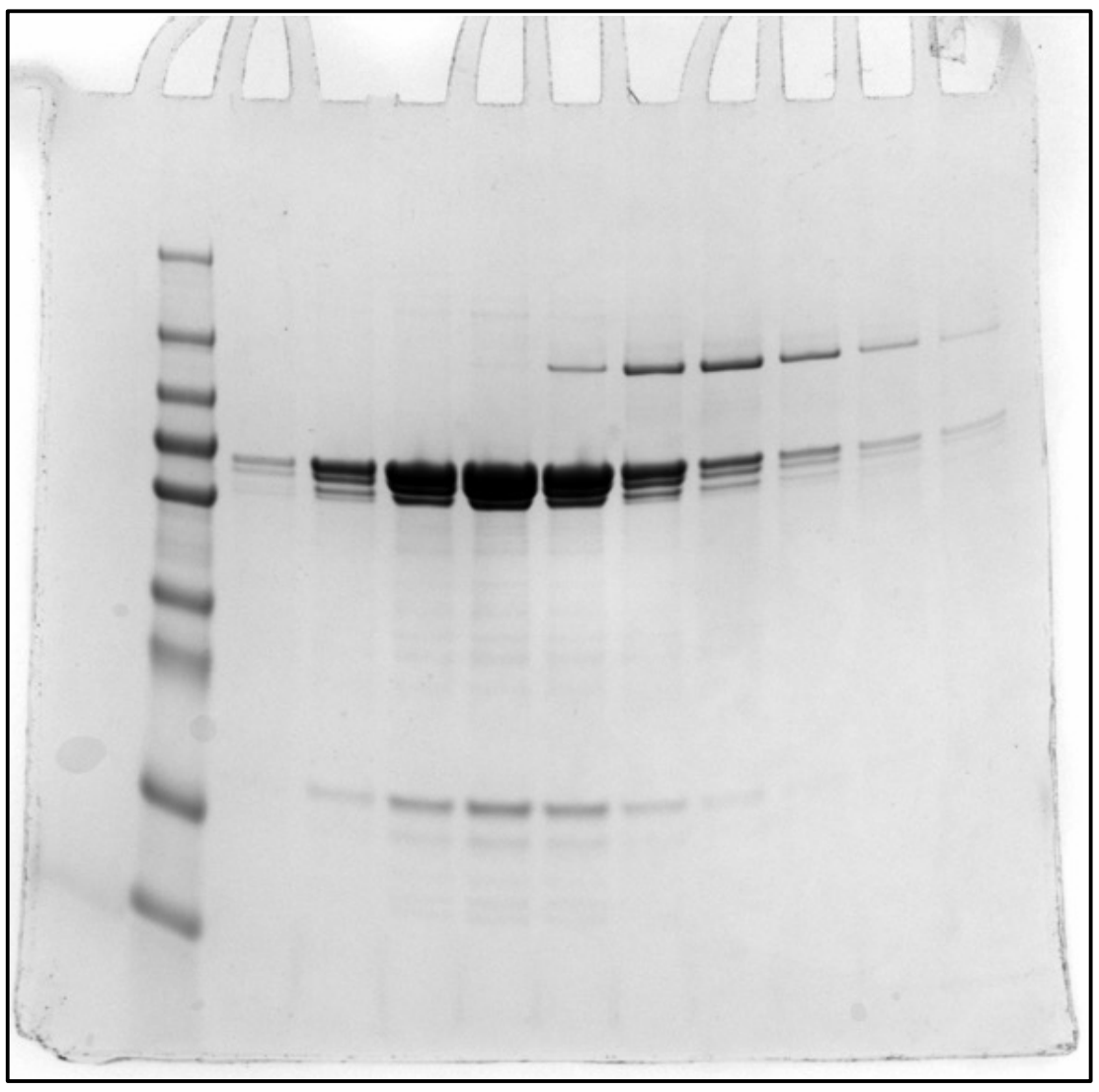

Fig. 1b uncropped

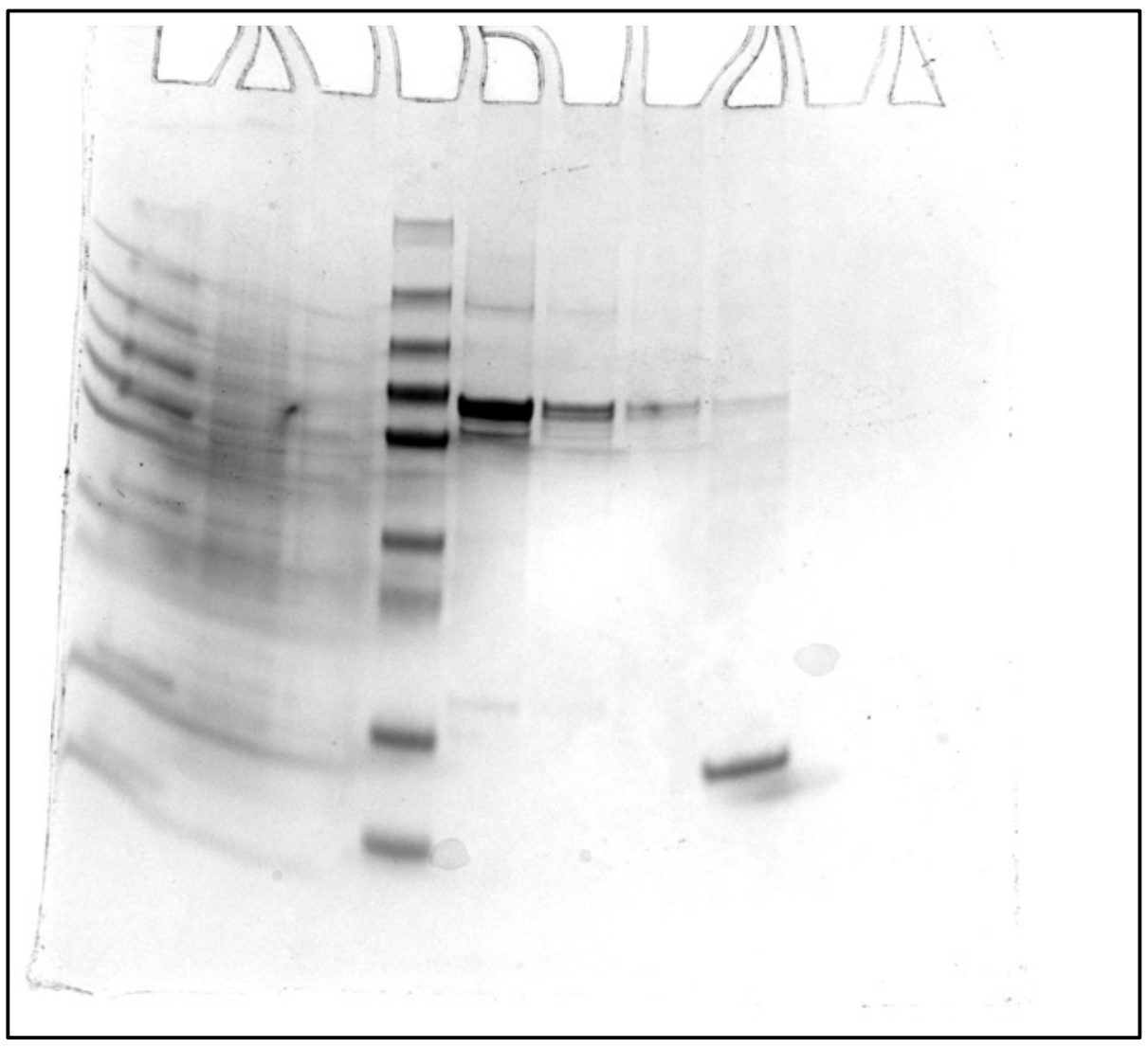

Fig. 1c uncropped

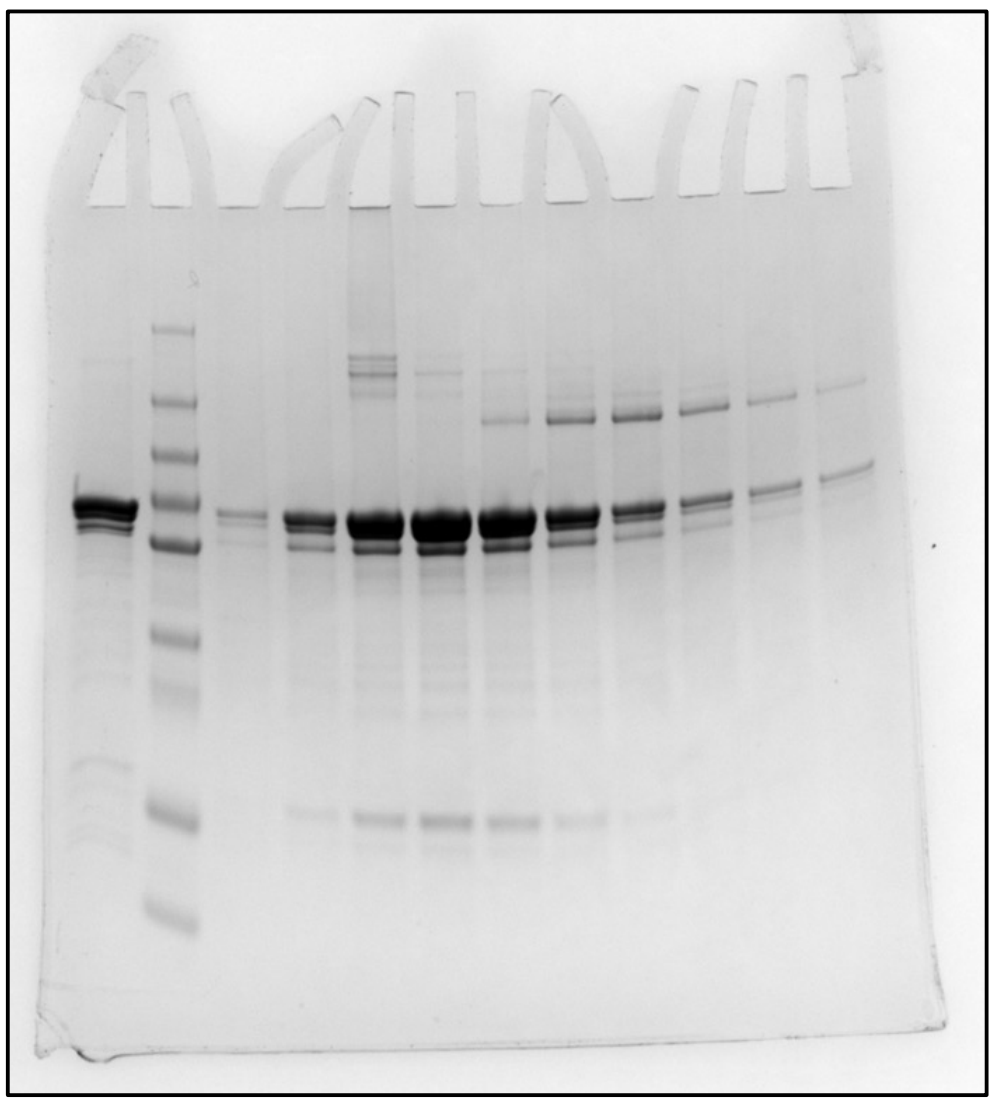

Fig. 1d uncropped

Supplementary Figure 5: Uncropped gels of Figure 1.
