# Supplementary material for: Architecture of an asymmetric short chain/long chain hybrid acyl‑CoA carboxylase from Mycobacterium smegmatis
Source: Commun Biol. 2026 Jun 11;9:1251. doi: 10.1038/s42003-026-10439-x (PMC13612425; doi:10.1038/s42003-026-10439-x)
Supplement: Supplementary file 3 [file 42003_2026_10439_MOESM3_ESM.pdf]

Description of additional supplementary file

File name: Supplementary Data 1

Description: Supplementary source data
