# Supplementary material for: Architecture of an asymmetric short chain/long chain hybrid acyl‑CoA carboxylase from Mycobacterium smegmatis
Source: Commun Biol. 2026 Jun 11;9:1251. doi: 10.1038/s42003-026-10439-x (PMC13612425; doi:10.1038/s42003-026-10439-x)
Supplement: Supplementary file 4 [file 42003_2026_10439_MOESM5_ESM.docx]

**Description of Additional Supplementary Files**

**File name:** A3D4D5Supplement210526R

**Description:** PDF document with the following supplementary tables and figures:

Supplementary Table 1. LC–MS/MS identification of ACCase subunits

Supplementary Figure 1: Cryo-EM data processing workflow for AccA3/AccD4/AccD5/AccE5 holo complex

Supplementary Figure 2: Cryo-EM data processing workflow for AccA3/AccD4/AccD5/AccE5 complex with propionyl-CoA

Supplementary Figure 3: Cryo-EM data processing workflow for AccA3/AccD4/AccD5/AccE5 complex with Arachidyl-CoA

Supplementary Figure 4: Local resolution estimation of AccA3/AccD4/AccD5/AccE5 complex without and with acyl-CoA substrates

Supplementary Figure 5: Uncropped gels of Figure 1

**File name:** Supplementary Data

**Description:** Excel file with the following source data sheets:

Figure_1b_SEC_profile

Figure_1d_SEC_profile

Figure_1e_C2CoA

Figure_1e_C2CoACO2

Figure_1e_C3CoA

Figure_1e_C3CoACO2

Figure_1e_C17CoACO2

Figure_1e_C18CoACO2
